# Phenotypic Switching Can Speed up Microbial Evolution

**DOI:** 10.1038/s41598-018-27095-9

**Published:** 2018-06-12

**Authors:** Andrew C. Tadrowski, Martin R. Evans, Bartlomiej Waclaw

**Affiliations:** 1 School of Physics and Astronomy, The University of Edinburgh, Peter Guthrie Tait Road, Edinburgh, EH9 3FD United Kingdom; 2 Centre for Synthetic and Systems Biology, The University of Edinburgh, Edinburgh, EH9 3FD United Kingdom

## Abstract

Stochastic phenotype switching has been suggested to play a beneficial role in microbial populations by leading to the division of labour among cells, or ensuring that at least some of the population survives an unexpected change in environmental conditions. Here we use a computational model to investigate an alternative possible function of stochastic phenotype switching: as a way to adapt more quickly even in a static environment. We show that when a genetic mutation causes a population to become less fit, switching to an alternative phenotype with higher fitness (growth rate) may give the population enough time to develop compensatory mutations that increase the fitness again. The possibility of switching phenotypes can reduce the time to adaptation by orders of magnitude if the “fitness valley” caused by the deleterious mutation is deep enough. Our work has important implications for the emergence of antibiotic-resistant bacteria. In line with recent experimental findings, we hypothesise that switching to a slower growing — but less sensitive — phenotype helps bacteria to develop resistance by providing alternative, faster evolutionary routes to resistance.

## Introduction

Biological evolution relies on two mechanisms which are instrumental in natural selection: preferential survival of better adapted individuals (selection) and variations among individuals (phenotypic variation). One of the sources of phenotypic variability is genetic alteration due to mutations and recombination. However, even genetically identical organisms will often behave differently because the same genotype can lead to many different phenotypes: the observable traits of an organism. This happens as a result of environmental factors and the organism’s history. Although ubiquitous and easily observed in animals and plants, phenotypic diversity can already be amply demonstrated in microorganisms. Examples range from different cell sizes depending on the growth medium^1^, through bistability in utilization of different food sources^2^, to diversification between motile/non-motile cells^3^. Microorganisms are often able to switch between these phenotypes in response to a change in external conditions such as the arrival of a new food source or depletion of the currently used one. A typical example is diauxic shift - a switch to another food source, for example from glucose to cellobiose in *L*. *lactis* when glucose becomes depleted^4^, which involves altering gene expression levels without changing the genetic code.

Some microorganisms switch seemingly randomly between two or more phenotypes even in the absence of external stimuli. This causes the population to become phenotypically heterogeneous. Several explanations have been proposed as to why *stochastic phenotype switching* has evolved^5^. One of them is the division of labour^6^ in which different microbial cells perform different functions, thus maximizing the benefit to the population. Another theory, called bet hedging^7,8^, proposes that in a fluctuating and unpredictable environment it pays to have a fraction of the population in a different state, which is perhaps maladapted to the present environment but better suited to possible future environments. Since only a small fraction of the population expresses the maladapted phenotype at any one time, this strategy conserves resources while allowing the population to stay prepared for an unexpected change. Examples include bacterial persisters^9–11^, flu(Ag43)/fim switch^9,12–14^ and competence to non-competence switching in the bacterium *B*. *subtilis*^15^. Animal cells also exhibit this behaviour. Epithelial-to-mesenhymal transition – a landmark in cancer progression – is thought to be a phenotypic, epigenetic change^16^. There is also some evidence that phenotypic switching may be involved in resistance to chemotherapy^17^, although this remains controversial^18^.

Here we investigate an alternative possible role for the evolution of phenotype switching in microbes: the facilitation of genetic evolution in a static environment. This question is a special case of a more general problem of the interplay between genetic and non-genetic (epigenetic) factors in biological evolution. Generic theoretical modelling (i.e. not specific to microorganisms) of this research question has a long history^19–23^. In particular, it has been pointed out that phenotypic variation can be advantageous for a population that is in a state far from the best-adapted genotype^20^. However, variation due to epigenetic mutations (switching phenotypes) can also slow down evolution if the distribution of epigenetic mutations is the same as the genetic ones due to an increased mutation load^24^.

Regarding microorganisms, one context in which non-genetic evolution has been studied is that of adaptation to a sudden change in the environment. Recent experiments on adaptation of unicellular algae to environmental stress have shown that reduction in epigenetic variation reduces the rate of evolution^25^. Bacterial persistence has also been addressed as a special case. Persistence is the ability of a subpopulation of genetically identical bacteria to survive high concentrations of an antibiotic^26^. Persistent cells often exist in the population before exposure to the antibiotic^11^ and are thus thought to be created through some form of stochastic phenotype switching. Theoretical population dynamics models of bacterial persistence have shown the existence of an optimal switching rate for environments that alternate between growth (no antibiotic) and stress (antibiotic) conditions^27^. It has been also suggested that persistence promotes evolution by increasing the rate of mutation and switching on mechanisms that decrease sensitivity to drugs^28,29^.

Previous theoretical work^30^ has demonstrated that switching to a persistent phenotype can provide a larger pool of bacteria in which to evolve genetic resistance to antibiotics. That work only addressed the situation in which resistance is caused by a single, beneficial mutation. However, a significant change of fitness, defined here as a measure of a microorganism’s growth rate, often requires multiple mutations. Although many fitness landscapes have at least one accessible pathway^31,32^, along which the fitness increases monotonically, in some cases a fitness valley — a genotype with a lower fitness than all its neighbours — is unavoidable. For example, developing resistance to the antibiotic streptomycin involves a fitness cost which must be counteracted by compensatory mutations, i.e. subsequent mutations that increase the microorganism’s fitness to at least that of the ancestral strain^33–35^.

Here we study evolution in the presence of such a fitness valley, in which a population must acquire two mutations to reach the best-adapted genotype. The first mutation is deleterious and corresponds to a valley in the fitness landscape that is crossed by a second, compensatory mutation. Theory and computer modelling suggests that fitness valleys significantly affect evolution^36–39^, often, but not always, slowing it down. However, in our model cells can also switch to an alternative phenotype in which the fitness landscape is flat. We show that this switching enables the population to avoid the costly valley, even if the alternative phenotype is less fit than the initial state. We also demonstrate the existence of an optimal range for the switching rate. In this range the time to evolve the best-adapted genotype can be reduced by many orders of magnitude compared to the case without switching. Finally, we show that if the switching rate is allowed to evolve it will converge to values within this optimal range.

## Model

**Figure 1:**
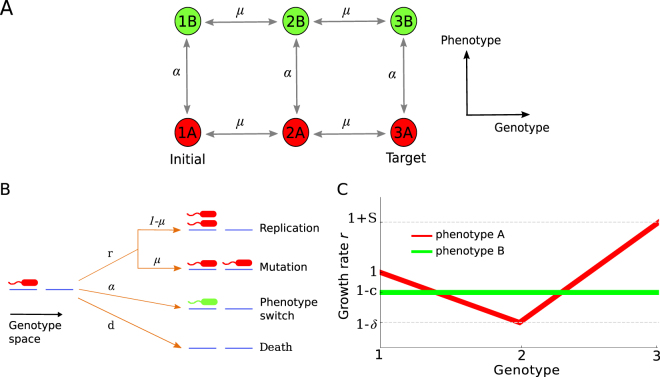
The model. (**A**) Diagram showing the six possible states of a cell and the available transitions between them. The genotypes are labelled 1, 2 and 3, the phenotypic states are labelled A and B. Transitions between genotypes/phenotypes occur at rates *μ* and *α*, respectively. All cells are initially in state 1A. Evolution continues until a single cell reaches the target state 3A. (**B**) Each cell can replicate, switch phenotype, or die with rates *r*, *α* and *d* respectively. Upon replication a cell has the probability *μ* of producing a mutant of each neighbouring genotype. (**C**) The fitness landscapes for both phenotypes. Phenotype A has a fitness valley at 2A while phenotype B has uniform fitness across all genotypes.

We consider a population of haploid cells. Each cell can be in one of two phenotypic states, A and B, and can assume one of three genotypes (“genetic states”), as shown in Fig. 1A,B. Cells replicate stochastically with rate *r*_*i*_(1 − *N*/*K*), where *i* labels one of the six possible states, *N* is the total population size and *K* is the carrying capacity of the environment. The logistic-like factor (1 − *N*/*K*) causes growth to cease when *N* becomes as large as *K*, which limits the total population size. During replication a cell will produce a mutated offspring of an adjacent genotype with probability *μ*. Mutation does not change the phenotypic state. All cells switch randomly between the states A and B at the symmetric switching rate *α*. Cells are randomly removed from the population at a constant death rate *d*. If the carrying capacity *K* is small ($K=10\cdots {10}^{4}$) the model is appropriate to describe a small microbial population growing in a microfluidic chemostat with constant dilution rate^40^. For larger *K* ($K={10}^{4}\cdots {10}^{9}$) the model is relevant to populations cultured in mesoscopic (cm-size) chemostats.

The population initially consists of all individuals of type 1A, i.e. of genotype 1 in phenotypic state A, which has the growth rate *r*_1A_ = 1 (arbitrary units). The initial population size, unless otherwise stated, is equal to the equilibrium size (1 − *d*)*K*. State 3A is the global maximum of both fitness landscapes with the growth rate *r*_3A_ = 1 + *S*, where *S* > 0 is its selective advantage over the “wild-type” 1A (Fig. 1C). Such a beneficial mutant has the probability of fixation, from a single individual in a population of cells in state 1A, approximately given^41^ by $\frac{1-{e}^{-S}}{1-{e}^{-KS}}\approx S$ when $1/K\ll S\ll 1$. We are interested in the *mean adaptation time T* (number of generations) that it takes for the population to evolve the first individual in state 3A, conditioned on the population not going extinct (otherwise the time would be infinite).

We shall begin by considering the case in which the growth rate *r*_2A_ = 1 − *δ*, while the growth rates *r*_1B_ = *r*_2B_ = *r*_3B_ = 1, which leads to a “fitness valley” in phenotype A and a flat fitness landscape in phenotype B (Fig. 1C). In the absence of phenotype switching the only way a population in state 1A can evolve an individual in state 3A is to go through the low-fitness state 2A. However, phenotype B allows the population to traverse an alternative route that avoids state 2A.

## Results

### An optimal range exists for the switching rate

**Figure 2:**
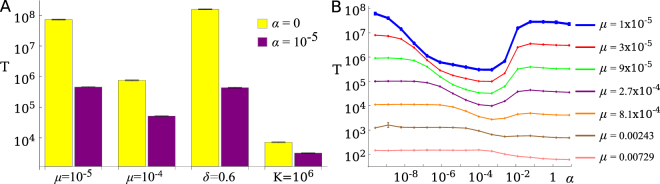
Mean adaptation time *T* for different scenarios – note logarithmic scale. (**A**) A bar chart comparing pairs of *T* values with and without switching phenotypes (*α* = 10^−5^ and *α* = 0 respectively) for different parameter values. Left-most pair of bars: *K* = 100, *μ* = 10^−5^, *δ* = 0.4 and *d* = 0.1. A label underneath each pair of bars indicates which variable has been changed compared to the left-most pair. (**B**) *T* as a function of the switching rate *α* for a range of mutation probabilities *μ*. Parameters as in (A). For small enough *μ* an optimal (minimizing *T*) switching rate can be seen.

Figure 2A shows simulation results examining the effect of phenotype switching on the mean adaptation time *T* for a range of the parameters of the model. In all cases the presence of low frequency switching, i.e. small *α*, decreases the mean adaptation time by orders of magnitude. Holding all other parameters fixed and changing only the switching rate reveals an optimal range for *α* (Fig. 2B) in which *T* is minimized provided *μ* is sufficiently small. For the smallest mutation probability (*μ* = 10^−5^) considered in Fig. 2B the minimal mean adaptation time is approximately two orders of magnitude lower than in the absence of switching (*α* = 0), or when switching is frequent (*α* very large). Only if the mutation rate is unrealistically large does switching not speed up evolution (Fig. S1). We shall see later that this behaviour is also typical at much larger carrying capacities *K* ~ 10^9^.

If switching rates are asymmetric, i.e. if the switching rates A → B and B → A are not the same, the mean adaptation time can still be significantly reduced through phenotype switching (Fig. S2). Also, in the absence of transitions between states 2A and 2B (i.e. removing this step from the model in Fig. 1A), the optimal *α* range of Fig. 2 disappears and the mean adaptation time decreases monotonically with *α* (Fig. S3).

### Fastest evolutionary trajectories avoid the valley

**Figure 3:**
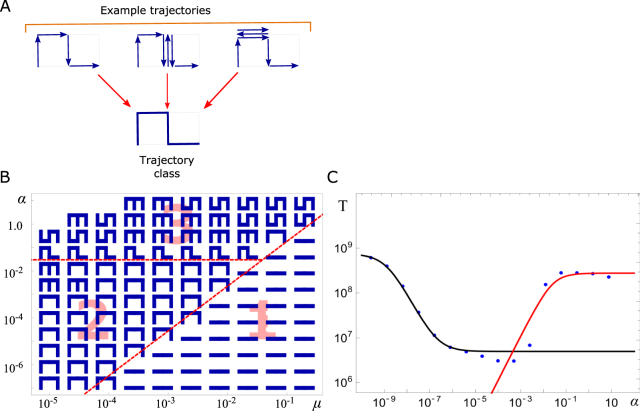
Trajectories of successful cells in genotype/phenotype space. (**A**) Diagram showing how different trajectories that go through the same set of states are grouped together into the same trajectory class. This class is represented as a symbol in which blue lines correspond to transitions made by the successful cell. (**B**) The most probable trajectory class as a function of *μ* and *α*, for *K* = 100, *δ* = 0.4 and *d* = 0.1. Three regions labelled 1, 2, 3 can be distinguished. (**C**) Comparison between analytic formula for the mean adaptation time (lines) and computer simulations (blue points). Parameters: *K* = 100, *μ* = 10^−5^, *d* = 0.1, *δ* = 0.4. Black curve corresponds to *T* = *T*_*A*_*T*_*B*_/(*T*_*A*_ + *T*_*B*_) (see SI Sec. X for explanation) with *T*_*A*_ given by Eq. (1) and *T*_*B*_ by Eq. (2). Red curve is from Eq. (3).

To understand the evolutionary trajectory selected in the optimal range of switching frequency we examined the histories of successful cells, i.e. the states from Fig. 1A visited during evolution from state 1A to the final state 3A. We then represented each cell’s trajectory in the state space as a sequence of links connecting the visited states (Fig. 3A). These were then classified as one of 21 classes by grouping sequences with multiple (back-and-forth) transitions between the states together with those without (Fig. 3A).

Figure 3B shows the most probable trajectory as a function of *μ* and *α*. As expected, simulated evolution favours different trajectories depending on the values of *μ*, *α*. The (*μ*, *α*)-space can be approximately separated into three regions, each corresponding to one or more trajectory classes. Region 1 corresponds to trajectories that go through the fitness valley at 2A. In this region mutations are frequent enough to offset the loss of fitness incurred when passing through the valley. In region 2 the dominant trajectory avoids the valley by switching to phenotype B. This region corresponds to the fastest adaptation times from Fig. 2B. Region 3 is characterised by a mixture of trajectory types that use both phenotype B and visit state 2A. This region corresponds to large *α* which not only enables transitions to phenotype B but also from state 2B to the deleterious state 2A. The latter is responsible for the increase in the mean adaptation time for large *α* in Fig. 2B since in this region states 2A and 2B behave as a combined state with fitness lower than that of state 2B (SI section VI). A similar decomposition into three regions can be obtained from the probability of individual transitions occurring between the states of the system (Fig. S4). Note that we focused here on small carrying capacities (*K* ~ 100) which enabled us to run many simulations and accurately determine “typical” trajectories. However, simulations for *K* up to 10^9^ showed very similar behaviour.

We can understand the origin of different regions in Fig. 3B by calculating the mean adaptation time *T* for trajectories going through or avoiding the valley analytically, in the limit of small *K*, *μ* (SI, section X). We calculate *T* along different trajectories for different ranges of *α*. For a given *α*, the system is most likely to follow a trajectory with the smallest *T*. This approach leads to the following results. When $\alpha \ll {\alpha }^{\ast }=K{\mu }^{2}d$, the population does not switch phenotypes and evolution goes through the valley. The mean adaptation time *T* is approximated by (SI, Eq. (S30))

1 $${T}_{A}\approx \frac{1}{(1-d)K{\mu }^{2}d(1/\delta -1)}.$$

This expression is valid in the part of region 1 from Fig. 3B far from the boundary between regions 1 and 2. As *α* increases and approaches *α*_−_ = *μd*, switching becomes more important. *α*_−_ corresponds to the boundary between regions 1 and 2. The mean adaptation time for such intermediate *α* is (SI, Eq. (36))

2 $${T}_{B}\approx \frac{1}{\alpha }+\frac{5}{\mu d}.$$

For $\mu d\ll \alpha \ll {\alpha }_{+}=d/K$, the system is in the optimal switching regime. The population evolves almost exclusively along trajectory 1A → 1B → 2B → 3B → 3A, avoiding fitness valley at state 2A. The mean adaptation time is minimal and equals $T\approx \frac{5}{\mu d}$ as follows from Eq. (2). The minimal time *T* depends only on the mutation rate *μd* – this is intuitive since mutations are expected to be the limiting step in this region. As *α* increases even further, trajectories including both states 2B and 2A become more likely. The horizontal boundary between region 2 and region 3 (mixed trajectories) in Fig. 3B is given by *α* ≈ *d*/*K*. In the large-*α* limit, the mean adaptation time is (SI, Eq. (S40))

3 $${T}_{{\rm{comb}},{\rm{ST}}}\approx \frac{1}{(1-d)K{\mu }^{2}d(1/(1-{r}_{2,{\rm{comb}}})-1)},$$

where *r*_2,comb_ is the effective fitness of genotype 2 (phenotypes A and B combined, Eq. (S39)). Figure 3C shows that Eqs (1, 2, 3) agree very well with the mean adaptation time obtained in computer simulations from Fig. 2B.

**Figure 4:**
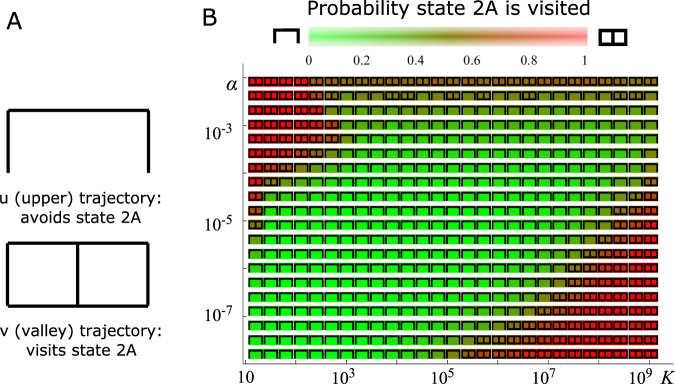
(**A**) Trajectories of successful cells can be grouped regarding whether they avoid (trajectory “u”) or visit (trajectory “v”) the fitness valley at state 2A. (**B**) The most common trajectory group and the probability of a trajectory visiting state 2A (colours, see the colour bar) as a function of switching rate *α* and carrying capacity *K*, for *μ* = 10^−6^. In all simulations *δ* = 0.4 and *d* = 0.1.

We next examined which trajectories would be preferred if we varied *K* instead of *μ*, as the carrying capacity is more likely to vary significantly in real microbial populations and is much easier to control experimentally than the mutation rate. Since we ascertained earlier (cf. the previous paragraph) that the time to adaptation is most significantly affected by whether the trajectory visits state 2A or not, we further reduced the number of trajectory classes to just two (Fig. 4A): the “u” (upper) trajectory type that avoids state 2A or the “v” (valley) trajectory type that visits it. We found a large region in the space (*K*,*α*) that favours trajectories that avoid the state 2A (Fig. 3B). This region extends to large values of *K* ~ 10^9^. The mean adaptation time depends non-monotonically on the population size *K* (Fig. S5); similar non-monotonic behaviour has been seen in models without phenotype switching^42,43^.

### Results are robust to small fitness costs and differences in the mutation rates of phenotypes A and B

**Figure 5:**
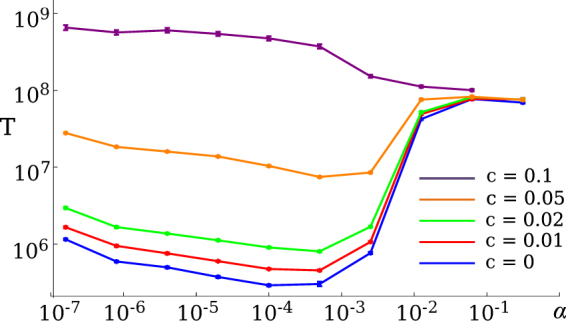
Mean adaptation time *T* plotted versus the switching rate *α*, for different fitness costs *c* = {0, 0.01, 0.02, 0.05, 0.1} of phenotype B and for parameters *K* = 100, *μ* = 10^−5^, *δ* = 0.9 and *d* = 0.1.

So far we have assumed that the alternative phenotype B has the same fitness as the wild-type state 1A. We shall now consider the case in which phenotype B is less adapted to the environment than the state 1A. This is a common scenario; for example, phenotypes more resistant to antibiotics often have a lower growth rate than susceptible phenotypes do in the absence of the drug^9^. To model this, we assume that the growth rates *r*_1B_ = *r*_2B_ = *r*_3B_ = 1 − *c*, where *c* > 0 is the fitness cost of switching to phenotype B (see Fig. 1C). A cell that switches from 1A to 1B grows more slowly but switching can still be advantageous if the alternative route by phenotype B is faster than crossing the fitness valley at state 2A. Figure 5 shows the mean adaptation time *T* as a function of switching rate *α* for different fitness costs. For sufficiently small *c* the same qualitative behaviour as for *c* = 0 is observed.

The same optimal switching rate range as in Fig. 2 can be seen in Fig. 5 for costs *c* ≤ 0.05 of state 1A’s fitness, although it reduces in significance as *c* increases. This is due to the increased time taken by trajectories that avoid state 2A as a result of the reduced growth rates of the phenotype B states. For large *c*, such as *c* = 0.1 in Fig. 5, there is no optimal switching range but the mean adaptation time *T* decreases monotonically with *α*. Stochastic phenotype switching thus speeds up evolution even in the presence of (moderate) fitness costs.

An optimal switching rate exists also in the case in which phenotypes A,B have different mutation rates. This may be the case for some antibiotics^44^ which induce a stress response characterized by a significant increase of the mutation rate^45^. To see how this would affect the results from Fig. 2, we performed simulations of the model with mutation probabilities *μ*_*A*_ = 10^−5^ for phenotype A and *μ*_*B*_ = 10^−5^, 10^−4^, 10^−3^ for phenotype B (SI, section VII). We observed that the range of optimal *α* values shifted to larger *α*’s and the depth of the valley decreases, as we increased *μ*_*B*_ (Fig. S7). However, even for *μ*_*B*_ = 100*μ*_*A*_ it was still disadvantageous for the population to switch phenotypes too fast (*T* increasing for large *α* in Fig. S7).

### Allowing the switching rate to evolve

**Figure 6:**
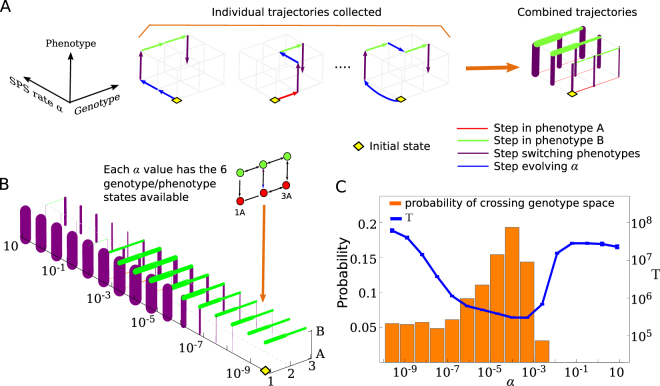
Evolution selects switching rates within the optimal range. (**A**) Left: examples of evolutionary trajectories in the extended model in which the stochastic phenotype switching (SPS) rate *α* also evolves. (**A**) Right: trajectories are used to calculate transition probabilities between the states of the system, which are then represented by the thickness of links connecting the states. Red links correspond to mutations in phenotype A, green links to mutations phenotype B and purple links to switching between phenotypes. (**B**) Graph of transition probabilities where line thicknesses are proportional to the probability that a successful trajectory will take that step. The parameters are *K* = 100, *μ* = 10^−5^, *δ* = 0.4 and *d* = 0.1, the same that were used in Fig. 2B (blue line). The population begins at the wild-type 1A with *α* = 2.56 × 10^−10^ and evolves until a cell in state 3A is produced. (**C**) The probability that genotype space is crossed at a given *α* in either phenotype A or B. The probability has a maximum where the mean adaptation time *T* for fixed *α* (plot from Fig. 2B superimposed on the same graph) has its minimum.

So far we have shown that there often exists an optimal range for the switching rate that results in the shortest mean adaptation time. This range coincides with successful cells avoiding the deleterious state 2A by switching to phenotype B. To see whether a variable switching rate would evolve to be in this optimal range, thus optimising the evolutionary process, we simulated a population of cells that started at 1A with *α* ≈ 0 initially. The switching rate was subsequently allowed to evolve: during replication, offspring were assigned a new *α* value with probability *μ*, the same as for mutations between genotypes 1 ↔ 2 ↔ 3. The new *α* was randomly and uniformly selected from a fixed set of possible values (*α* = 2.56 × 10^−10^ × 5^*i*^ for integer *i* ∈ [0, 15]); similar results were obtained when *α* evolved incrementally (Fig. S8). In this extended model the evolutionary trajectory of successful cells spans three dimensions: two for the genotype, one of which evolves the switching rate, and one for the two phenotypes A and B (Fig. 6A). The end point of the trajectory remains a cell produced at state 3A regardless of its switching rate. We collected trajectories of successful cells in the expanded state space, including states with different *α*, and calculated transition probabilities between any two states. Figure 6B shows these probabilities as links of different thickness between the states in state space. We observe that successful trajectories are unlikely to feature genotype mutations in phenotype A (very few red links). Instead, most successful cells first switch to state 1B, mutate to 3B, and switch back to state 3A. The evolved switching rate often falls within the optimal range found in Fig. 2B for the same set of parameters *K*, *μ* and *d*. This is further illustrated in Fig. 6C where we overlay the mean adaptation time from Fig. 2B with a bar chart that shows the probability that a particular *α* was selected by evolution. See also SI Sec. IX, Fig. S9 for separate plots for mutations 1 → 2 and 2 → 3.

Figure 6C reveals two more interesting features. First, we see that cells that have evolved *α* above the optimal range (*α* ≥ 0.02) are unlikely to be successful due to fast transitions between states 2B and 2A (fitness valley). Such transitions effectively lower the fitness of state 2B and create a new fitness valley at this state (SI, section VI). Second, we see that a large number of trajectories cross genotype space at *α* values below the optimal *α* range. However, the plot of *T* in Fig. 6C suggests this will be a slow process. The latter observation can be explained if we assume these cells first evolve a large switching rate, allowing them to switch quickly to phenotype B, before their switching rate decreases again. This hypothesis is corroborated by observing that at low *α* there is little phenotype switching (purple links in Fig. 6B) and yet the likelihood of mutations in phenotype B across genotype space is large (green links in Fig. 6B). This provides an alternative efficient trajectory for a population to cross the fitness valley. However, such trajectories need at least one extra mutation compared to those that evolve *α* directly to within the optimal range. Therefore evolution is dominated by these latter trajectories, in particular for low mutation probabilities *μ*.

## Discussion

Phenotypic plasticity has been recently shown to affect Darwinian evolution of animals^46^. Our theoretical research suggests that stochastic phenotype switching is also able to significantly speed up evolution of microbes, perhaps by many orders of magnitude. Switching phenotypes enables cells to evade a fitness valley which may be difficult to cross otherwise, particularly for small populations. Although our model is a mathematical idealisation and it has only three genotypes, we believe the findings presented here are much more general and apply to realistic, multi-dimensional fitness landscapes^31,47,48^. Epistasis causes such landscapes to contain many local fitness maxima separated by genotypes with lower growth rate^49^. In a small population, in the absence of phenotype switching, evolution can spend much time in a local maximum before “tunnelling” through one of the fitness valleys. Phenotype switching is equivalent to having a second fitness landscape in which minima and maxima can be located at different genotypes compared to the first landscape. Phenotype switching may therefore provide alternative routes between otherwise isolated fitness maxima in both landscapes and enable evolution to proceed faster. The mechanisms explored here, in a similar way to the proposed interplay between genetic and epigenetic mutations^23^, add another dimension to the complexity of evolutionary pathways^31,48,50^ which is relevant for the predictability of evolution^51^.

We now discuss how our computational results can be used to predict the behaviour of real microbial populations. The mechanism presented here is relevant if (i) the switching rate is sufficiently fast, (ii) an alternative phenotype exists in which the fitness valley is significantly more shallow or does not exist and (iii) the population is initially trapped in a local fitness maximum. Let us first discuss point (i). In microbes, the probability of single nucleotide mutation is 10^−10^–10^−9^ per replication^52,53^ but can be higher (~10^−7^) in mutator strains^53,54^ or in the presence of antibiotics^55^. Assuming *μ* = 10^−6^ which would correspond to ~10 point mutations in a mutator strain or any loss-of-function mutation in a non-mutator strain (assuming a typical gene length 1 kbp^56^), Fig. 4B indicates that phenotype switching would be the preferred mode of evolution for *α* < 10^−4^ in a population of *K* = 10^3^ cells, for 10^−7^ < *α* < 5 × 10^−3^ for intermediate size *K* = 10^6^, and for *α* ≈ 10^−3^ for *K* = 10^9^. For lower mutation probabilities *μ* we expect this behaviour to extend to lower switching rates, consistent with the increasing optimal ranges seen in Fig. 2B, while maintaining approximately the same upper limit of *α* ≈ 10^−3^–10^−2^. Switching rates encountered in nature can be as large as this^13,14^ and thus stochastic phenotype switching could be a significant force in the biological evolution of microbes.

To discuss points (ii) and (iii) we focus on antibiotic resistance evolution because fitness effects of specific point mutations are particularly well documented for many drugs. We already mentioned streptomycin as an example drug in which a fitness valley can occur when a population of microbes is exposed to sub-lethal concentrations of the antibiotic. In this scenario, state 1A would correspond to a ‘wild-type’ genotype whose growth rate is slightly lowered by the presence of the antibiotic, but cells are still able to reproduce. The fitness landscape (Fig. 1C) for phenotype A would then correspond to a situation in which an initial mutation lowers the growth rate by e.g. making the target enzyme less susceptible to the drug but also less efficient^33,34,57^. A second mutation compensates for the loss of efficiency and increases the growth rate beyond that of the ancestral strain. In the same example the flat landscape for phenotype B would result from switching to a state with higher gene expression of multidrug efflux pumps or antibiotic-degrading enzymes. This has been observed for a biofilm-forming bacterium *P*. *aeruginosa* which can switch to a resistant phenotype in the presence of antibiotics^58,59^. Alternatively, phenotype B can be a persister; although the conventional view is that persistent cells do not replicate, this has been recently challenged^60^. Slower (but non-zero) replication rates can enable persisters to mutate and explore a larger set of genotypes than the non-persister (phenotype A) would be able to do in the absence of switching.

We stress that, according to our model, an increased rate of evolution in the presence of phenotype switching is caused by avoiding the fitness valley by following a flatter fitness path allowed by switching phenotype. This is qualitatively different to another possible mechanism in which switching to an alternative phenotype with a higher mutation rate^45^ increases the rate of valley crossing.

Although our model is not aimed at describing a particular experimental system, in terms of the number of relevant states and transitions between them, recent work suggests that mechanisms similar to those in our model may be at play in real microbial populations. In particular, references^ 44,61^ show that the bacterium *E*. *coli* switches to a filamentous phenotype (our phenotype B) following the SOS response when exposed to sub-inhibitory concentrations of the antibiotic ciprofloxacin. These filamentous cells occasionally produce normal-length cells (our phenotype A) which are resistant to ciprofloxacin. It has been hypothesized that phenotype B provides a safe niche that gives bacteria enough time to evolve resistance. Our interpretation of the results of these experiments is that the increase in resistance caused by phenotypic mechanisms makes the fitness landscape more flat, albeit probably not in exactly the same way as in our model. Importantly, the switching between phenotypes in this example is not entirely random but a response to stress, and the mutation rate increases as well. However, the observed behaviour is similar to that predicted by our model. We may thus expect that a model similar to the one presented here but with different and perhaps time-dependent transitions between genotypes and phenotypes could be used to describe the results of the experiments from references^44,61^.

For the above scenario of the evolution of resistance to ciprofloxacin, even though the mechanistic details may not be identical to that of our model, we can still try to make qualitative but experimentally falsifiable predictions. For example, we can predict that, in the absence of switching (no SOS response/filamentous cells), the evolution of resistance would be much slower. The same effect should be observed if, instead of blocking the SOS response, filamentous cells were removed from the population. This could be achieved experimentally by growing cells in a chemostat-like device with continuous removal of cells; non-replicating filamentous cells would be quickly flushed away and no or very slow evolution of resistance would be observed. If filamentous cells were instead kept in the reaction vessel (e.g. by trapping them in tiny channels in a microfluidic device such as the “death galaxy”^61^), evolution should proceed much faster.

### Addendum

While this article was in revision, a research had been published^62^ which experimentally showed that tolerance to low-level antibiotics that develops in a sub-population of persisters facilitates further genetic evolution. Even though evolution in the described work occurs not in a static environment but following cyclic exposure to antibiotics, this work provides further support for general mechanisms outlined in the present manuscript.

## Methods

Custom written Java programs were used to simulate the model. For smaller *K* (*K* ≤ 100) each cell was assigned a current type (one of the 6 possible states from Fig. 1A) and a sequence of past states beginning at state 1A. A variant of the kinetic Monte Carlo algorithm^63^ was used to evolve the system. In each time step, a random cell and action (replication, death, or phenotype switch) was selected with a probability proportional to the rate with which that action occurs. A second random number drawn from an exponential distribution, with mean equal to the inverse of the total rate of all possible actions in the system, was used as a measure of time elapsed during that step. For larger *K* > 100 (or when *c* > 0) the evolution of the system was approximately modelled using a type of *τ*–leaping algorithm^64^.

Statistical analysis was performed in Mathematica using a custom-written code. Mean values presented in all plots were obtained by averaging over 10^4^ runs, except for Fig. 4B where points were averaged over 10^2^–10^3^ runs. Error bars are s.e.m. (standard error of the mean). The mean adaptation time is expressed in units 1/*d* which correspond approximately to the time it takes for all cells to replicate (“one generation”).

## Electronic supplementary material

Supplementary Material

## References

[1] Yao Z, Davis RM, Kishony R, Kahne D, Ruiz N (2012). Regulation of cell size in response to nutrient availability by fatty acid biosynthesis in Escherichia coli. Proceedings of the National Academy of Sciences.

[2] Ozbudak EM, Thattai M, Lim HN, Shraiman BI, van Oudenaarden A (2004). Multistability in the lactose utilization network of Escherichia coli. Nature.

[3] Kearns DB, Losick R (2005). Cell population heterogeneity during growth of Bacillus subtilis. Genes & Development.

[4] Solopova A (2014). Bet-hedging during bacterial diauxic shift. Proceedings of the National Academy of Sciences.

[5] Ackermann M (2015). A functional perspective on phenotypic heterogeneity in microorganisms. Nature Reviews Microbiology.

[6] Ackermann M (2008). Self-destructive cooperation mediated by phenotypic noise. Nature.

[7] Kussell E, Leibler S (2005). Phenotypic Diversity, Population Growth, and Information in Fluctuating Environments. Science.

[8] Beaumont HJE, Gallie J, Kost C, Ferguson GC, Rainey PB (2009). Experimental evolution of bet hedging. Nature.

[9] Balaban NQ, Merrin J, Chait R, Kowalik L, Leibler S (2004). Bacterial Persistence as a Phenotypic Switch. Science.

[10] Kussell E, Kishony R, Balaban NQ, Leibler S (2005). Bacterial Persistence A Model of Survival in Changing Environments. Genetics.

[11] Dhar N, McKinney JD (2007). Microbial phenotypic heterogeneity and antibiotic tolerance. Current Opinion in Microbiology.

[12] Hasman H, Schembri M, Klemm P (2000). Antigen 43 and type 1 fimbriae determine colony morphology of *Escherichia coli* K-12. Journal of bacteriology.

[13] van der Woude MW, Henderson IR (2008). Regulation and function of Ag43 (flu). Annual Review of Microbiology.

[14] Adiciptaningrum AM, Blomfield IC, Tans SJ (2009). Direct observation of type 1 fimbrial switching. EMBO reports.

[15] Maamar H, Dubnau D (2005). Bistability in the Bacillus subtilis K-state (competence) system requires a positive feedback loop: Bistability in B. subtilis competence. Molecular Microbiology.

[16] McDonald OG, Wu H, Timp W, Doi A, Feinberg AP (2011). Genome-scale epigenetic reprogramming during epithelial-to-mesenchymal transition. Nature Structural & Molecular Biology.

[17] Kemper K, de Goeje PL, Peeper DS, van Amerongen R (2014). Phenotype switching: tumor cell plasticity as a resistance mechanism and target for therapy. Cancer Research.

[18] Zapperi, S. & La Porta, C. A. M. Do cancer cells undergo phenotypic switching? The case for imperfect cancer stem cell markers. *Scientific Reports* **2** (2012). 10.1038/srep00441 PMC3369193 22679555

[19] Kirkpatrick M, Lande R (1989). The Evolution of Maternal Characters. Evolution.

[20] Pál C (1998). Plasticity, memory and the adaptive landscape of the genotype. Proceedings of the Royal Society of London B: Biological Sciences.

[21] Day T, Bonduriansky R (2011). A Unified Approach to the Evolutionary Consequences of Genetic and Nongenetic Inheritance. The American Naturalist.

[22] Geoghegan JL, Spencer HG (2012). Population-epigenetic models of selection. Theoretical Population Biology.

[23] Klironomos FD, Berg J, Collins S (2013). How epigenetic mutations can affect genetic evolution: Model and mechanism. BioEssays.

[24] Kronholm I, Collins S (2016). Epigenetic mutations can both help and hinder adaptive evolution. Molecular Ecology.

[25] Kronholm I, Bassett A, Baulcombe D, Collins S (2017). Epigenetic and Genetic Contributions to Adaptation in Chlamydomonas. Molecular Biology and Evolution.

[26] Brauner A, Fridman O, Gefen O, Balaban NQ (2016). Distinguishing between resistance, tolerance and persistence to antibiotic treatment. Nature Reviews Microbiology.

[27] Patra P, Klumpp S (2013). Population Dynamics of Bacterial Persistence. PLoS One.

[28] Cohen N, Lobritz M, Collins J (2013). Microbial Persistence and the Road to Drug Resistance. Cell Host & Microbe.

[29] Harms A, Maisonneuve E, Gerdes K (2016). Mechanisms of bacterial persistence during stress and antibiotic exposure. Science.

[30] Levin BR, Rozen DE (2006). Non-inherited antibiotic resistance. Nature Reviews Microbiology.

[31] Weinreich DM (2006). Darwinian Evolution Can Follow Only Very Few Mutational Paths to Fitter Proteins. Science.

[32] Franke J, Klözer A, de Visser JA, Krug J (2011). Evolutionary accessibility of mutational pathways. PLoS computational biology.

[33] Schrag SJ, Perrot V (1996). Reducing antibiotic resistance. Nature.

[34] Schrag SJ, Perrot V, Levin BR (1997). Adaptation to the fitness costs of antibiotic resistance in Escherichia coli. Proceedings of the Royal Society of London. Series B: Biological Sciences.

[35] Maisnier-Patin S, Berg OG, Liljas L, Andersson DI (2002). Compensatory adaptation to the deleterious effect of antibiotic resistance in Salmonella typhimurium. Molecular Microbiology.

[36] Weissman D, Desai M, Fisher D, Feldman M (2009). The rate at which asexual populations cross fitness valleys. Theoretical population biology.

[37] Weinreich D, Chao L (2005). Rapid evolutionary escape by large populations from local fitness peaks is likely in nature. Evolution.

[38] Covert AW, Lenski RE, Wilke CO, Ofria C (2013). Experiments on the role of deleterious mutations as stepping stones in adaptive evolution. Proceedings of the National Academy of Sciences.

[39] Szendro IG, Franke J, Visser JAGM (2013). d. & Krug, J. Predictability of evolution depends nonmonotonically on population size. Proceedings of the National Academy of Sciences.

[40] Balagaddé FK, You L, Hansen CL, Arnold FH, Quake SR (2005). Long-Term Monitoring of Bacteria Undergoing Programmed Population Control in a Microchemostat. Science.

[41] Nowak, M. A. *Evolutionary Dynamics* (Harvard University Press, 2006).

[42] Sumedha, Martin OC, Peliti L (2007). Population size effects in evolutionary dynamics on neutral networks and toy landscapes. Journal of Statistical Mechanics: Theory and Experiment.

[43] Elena SF, Wilke CO, Ofria C, Lenski RE (2007). Effects of population size and mutation rate on the evolution of mutational robustness. Evolution.

[44] Bos J (2015). Emergence of antibiotic resistance from multinucleated bacterial filaments. Proceedings of the National Academy of Sciences.

[45] Cirz RT (2005). Inhibition of Mutation and Combating the Evolution of Antibiotic Resistance. PLoS Biol.

[46] Ghalambor, C. K. *et al*. Non-adaptive plasticity potentiates rapid adaptive evolution of gene expression in nature. *Nature* (2015). 10.1038/nature15256 26331546

[47] Szendro IG, Schenk MF, Franke J, Krug J, Visser JAGMD (2013). Quantitative analyses of empirical fitness landscapes. Journal of Statistical Mechanics: Theory and Experiment.

[48] Palmer, A. C. *et al*. Delayed commitment to evolutionary fate in antibiotic resistance fitness landscapes. *Nature Communications* **6** (2015). 10.1038/ncomms8385 PMC4548896 26060115

[49] Korona R, Nakatsu C, Forney L, Lenski R (1994). Evidence for multiple adaptive peaks from populations of bacteria evolving in a structured habitat. Proceedings of the National Academy of Sciences of the United States of America.

[50] Poelwijk FJ, Kiviet DJ, Tans SJ (2006). Evolutionary Potential of a Duplicated Repressor-Operator Pair: Simulating Pathways Using Mutation Data. PLoS Computational Biology.

[51] de Visser JAGM, Krug J (2014). Empirical fitness landscapes and the predictability of evolution. Nature Reviews Genetics.

[52] Drake JW (1991). A constant rate of spontaneous mutation in DNA-based microbes. Proceedings of the National Academy of Sciences.

[53] Lee H, Popodi E, Tang H, Foster PL (2012). Rate and molecular spectrum of spontaneous mutations in the bacterium Escherichia coli as determined by whole-genome sequencing. Proceedings of the National Academy of Sciences of the United States of America.

[54] Sniegowski PD, Gerrish PJ, Lenski RE (1997). Evolution of high mutation rates in experimental populations of *E. coli*. Nature.

[55] Gillespie SH, Basu S, Dickens AL, O’Sullivan DM, McHugh TD (2005). Effect of subinhibitory concentrations of ciprofloxacin on Mycobacterium fortuitum mutation rates. Journal of Antimicrobial Chemotherapy.

[56] Kerner MJ (2005). Proteome-wide analysis of chaperonin-dependent protein folding in *Escherichia coli*. Cell.

[57] Marcusson LL, Frimodt-Moller N, Hughes D (2009). Interplay in the selection of fluoroquinolone resistance and bacterial fitness. PLoS pathogens.

[58] Breidenstein EBM, de la Fuente-Núñez C, Hancock REW (2011). Pseudomonas aeruginosa: all roads lead to resistance. Trends in Microbiology.

[59] Drenkard E (2003). Antimicrobial resistance of Pseudomonas aeruginosa biofilms. Microbes and Infection.

[60] Wakamoto Y (2013). Dynamic Persistence of Antibiotic-Stressed Mycobacteria. Science.

[61] Zhang Q (2011). Acceleration of Emergence of Bacterial Antibiotic Resistance in Connected Microenvironments. Science.

[62] Levin-Reisman, I. *et al*. Antibiotic tolerance facilitates the evolution of resistance. *Science* eaaj2191 (2017). 10.1126/science.aaj2191 28183996

[63] Gillespie DT (1977). Exact stochastic simulation of coupled chemical reactions. The journal of physical chemistry.

[64] Gillespie DT (2001). Approximate accelerated stochastic simulation of chemically reacting systems. The Journal of Chemical Physics.

