# Supplementary material for: Phenotypic Switching Can Speed up Microbial Evolution
Source: Sci Rep. 2018 Jun 12;8:8941. doi: 10.1038/s41598-018-27095-9 (PMC5997679; doi:10.1038/s41598-018-27095-9)
Supplement: Supplementary file 1 — Supplementary Material [file 41598_2018_27095_MOESM1_ESM.pdf]

Phenotypic Switching Can Speed up Microbial Evolution
Supplementary Information

Andrew C. Tadrowski, Martin R. Evans, and Bartlomiej Waclaw

I. CONDITIONS FOR PHENOTYPIC SWITCHING TO SPEED UP EVOLUTION

In Fig. 2A we compared the mean adaptation time T with and without phenotypic switching for various parameters. In all cases switching decreased T. However, if the mutation probability μ is large enough, the pathway that goes directly through the valley genotype 2A may take less time than any pathway involving the alternative phenotype B. Figure S1 shows T as a function of mutation probability μ for the case with and without switching. For large enough μ, switching makes no observable difference to the mean adaptation time as successful cells do not use an alternative pathway. This is the same behaviour observed in region 1 of Fig. 3B.

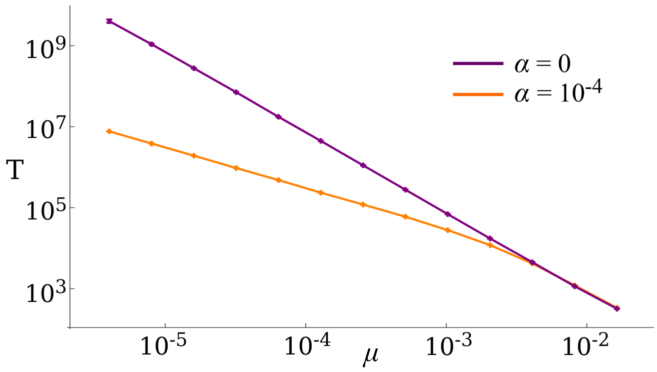

Figure S1: Phenotypic switching does not speed up evolution for large mutation probabilities μ. Shown is the mean adaptation time T from computer simulations as a function of μ with and without phenotype switching ($\alpha = 10^{-4}$ and $\alpha = 0$ respectively), for $K = 100, \delta = 0.4$ and $d = 0.1$.

II. ASYMMETRIC SWITCHING RATES

Figure S2 shows the mean adaptation time when the switching rates between the two phenotypes are asymmetric, α (A→B) and β (B→A), but identical for all genotypes. To create the plot we fixed the ratio α/β to $10^{-2}, \ldots, 10^2$ and varied α. For all but the largest ratio $\alpha/\beta = 10^2$ the mean adaptation time is minimized for a certain range of α.

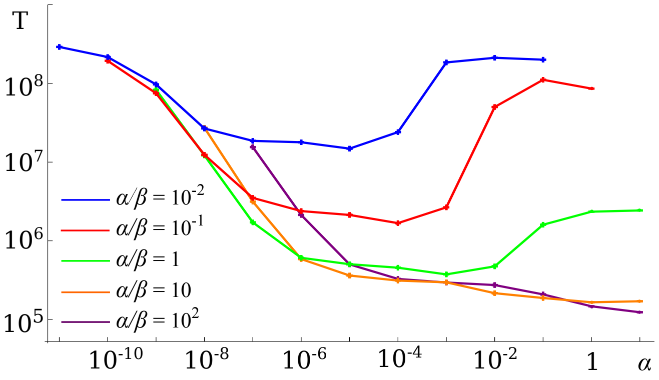

Figure S2: Mean adaptation time for different forward and backward switching rates α (A→B) and β (B→A). The parameters are $K = 20, \mu = 10^{-5}, \delta = 0.4, d = 0.1$.

III. MEAN ADAPTATION TIME DECREASES MONOTONOUSLY WITH α IN THE ABSENCE OF SWITCHING BETWEEN STATES 2A AND 2B

We identified that region 3 in Fig. 3B appears due to the existence of the step between states 2A and 2B (see Fig. 1A). For large α values a trajectory via phenotype B is likely to involve switching to the deleterious state 2A. This reduces the average fitness of the population and causes the mean adaptation time to increase with α (Fig. 2B). To demonstrate this consider the model without switching between states 2A and 2B (Fig. S3A). The mean adaptation time T as a function of switching rate α decreases monotonically in this modified model (Fig. S3B).

IV. PROBABILITY OF INDIVIDUAL STEPS FOR SUCCESSFUL TRAJECTORIES

In Fig. 3B we showed the most common trajectory classes for successful cells as a function of (μ, α). A more detailed picture can be obtained by looking at the probability that each step occurs in a successful cell's trajectory. For each pair of (μ, α) we create a diagram of transitions between states (i.e. 1A,1B,...,3A,3B) in which the thickness of each link corresponds to the probability that that step is taken (Fig. S4A). Figure S4B shows a graph in which diagrams for different pairs of (μ, α) have been put together. This graph is very similar to Fig. 3B. This confirms that the most-common trajectories plotted in Fig. 3B are good representatives of the "typical" trajectories of successful cells.

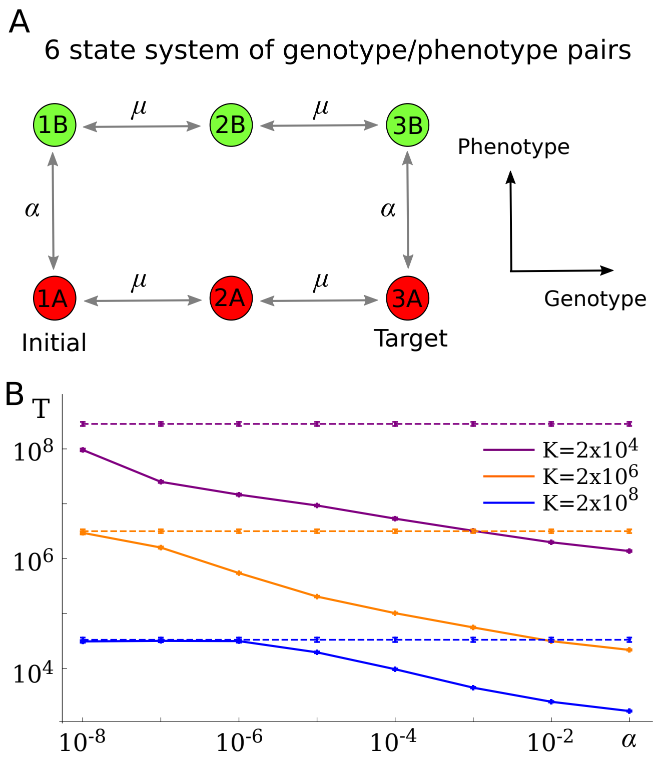

Figure S3: Mean adaptation time T without phenotype switching between states 2A and 2B. (A) The modified model. (B) T as a function of switching rate α for $K = 2\times10^4, 2\times10^6$ and 2×10^8. Dashed lines indicate T when $\alpha = 0$. Remaining parameters are $\mu = 10^{-6}$, $\delta = 0.4$ and $d = 0.1$

V. MEAN ADAPTATION TIME AS FUNCTION OF CARRYING CAPACITY

We can plot differently the same simulation data used in Fig. 4B to explore how the mean adaptation time T depends on the carrying capacity K. Figure S5 shows T for different values of the switching rate α. A non-monotonic behaviour can be seen: T is maximal for intermediate K and falls off for both small and large carrying capacities.

VI. THE EQUILIBRIUM DISTRIBUTION BETWEEN PHENOTYPES AT GENOTYPE 2

In the main text we posited that when α becomes too large the beneficial effect that state 2B has on the evolution of the population reduces, through its connection with the deleterious valley state 2A. To demonstrate this we consider here the equilibrium distribution that a population confined to genotype 2 will have between the states 2A and 2B. Such confinement at genotype 2 can be likened to the theoretical situation in which the mutation rate is much smaller (i.e. by orders of magnitude) than the rate of phenotype switching α. Therefore a population at genotype 2 can be assumed to reach its

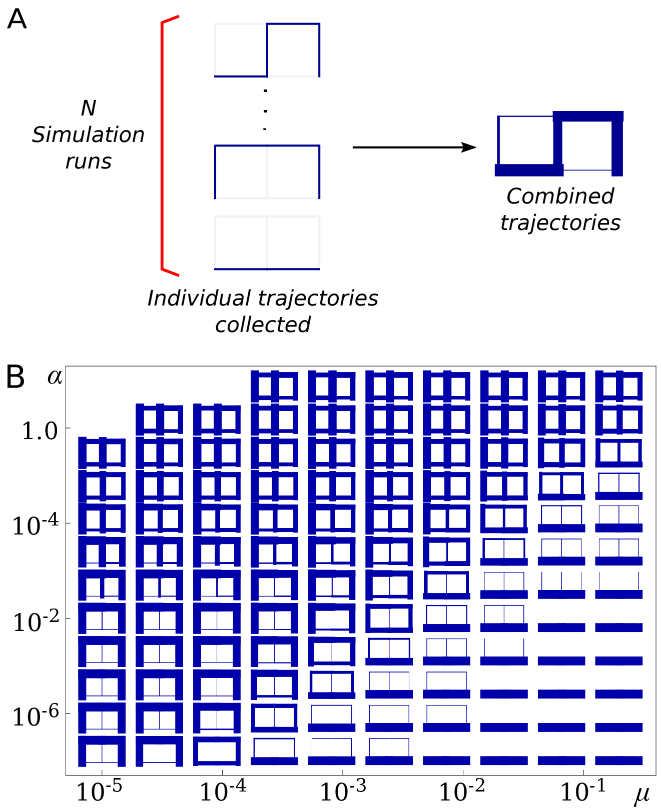

Figure S4: The probability of each step being taken by successful cells. (A) Constructing images that display the probability that each step is taken. By collecting many successful trajectory classes we can compile them into a single image that consists of links of different thicknesses, where the thickness of each link is proportional to the probability that that step is taken by a successful cell. (B) The probability each step is taken as a function of μ and α. Remaining parameters are $K = 100$, $\delta = 0.4$ and $d = 0.1$.

equilibrium distribution before the time it takes for the next mutation to occur. In this case the population at states 2A and 2B can be described by the following coupled differential equations

$$\dot{N}_{2A} = \xi N_{2A} r_{2A}(1-2\mu) + \alpha N_{2B} - \alpha N_{2A} - dN_{2A} \quad \text{(S1)}$$
$$\dot{N}_{2B} = \xi N_{2B} r_{2B}(1-2\mu) + \alpha N_{2A} - \alpha N_{2B} - dN_{2B} \quad \text{(S2)}$$

where N_{2A} and N_{2B} are the population number at states 2A and 2B respectively and $\xi = [1-(N_T/K)] \approx d$ is the logistic factor. These equations can be solved analytically to find the steady-state values of N_{2A} and N_{2B} as a function of α. The solution for the steady state distribution at state 2B, expressed as a frequency n_{2B} such that $n_{2B} = N_{2B}/(N_{2A}+N_{2B})$, is given by

$$n_{2B} = \frac{\delta d - (2-3\delta)\alpha + \sqrt{4\alpha^2(1-\delta) + (d+\alpha)^2\delta^2}}{2\delta(d+2\alpha)}. \quad \text{(S3)}$$

A plot of both n_{2B} from Eq. S3 and $n_{2A} = (1-n_{2B})$ are shown in Fig. S6. We can see that n_{2B} reduces

monotonically from 1 to the limiting value of 0.5 with increasing α. As this happens the average fitness of the entire population at genotype 2, defined as $\overline{r}_2 = n_{2A}r_{2A} + n_{2B}r_{2B}$, will decrease and it is straightforward to show that

$$\overline{r}_2 \to 1 - \frac{\delta}{2} \quad \text{as} \quad \alpha \to \infty. \tag{S4}$$

Therefore in the limit of large α the equilibrium distribution of the population is an equal distribution between states 2A and 2B. In this limit the selective differences between states 2A and 2B are not experienced by the population and the two phenotype system maps to a single phenotype problem in which the fitness of genotype 2 is equal to the $\overline{r}_2$ limit given in Eq. S4.

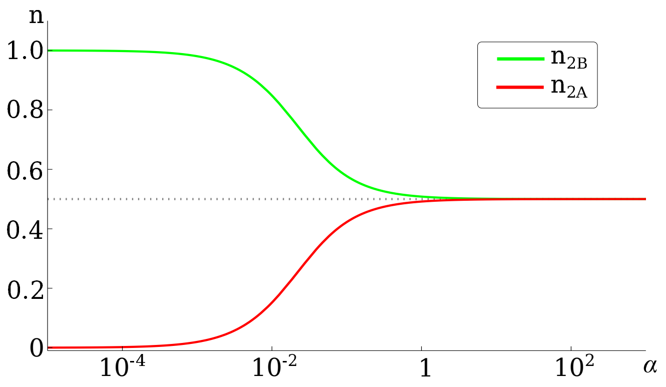

Figure S6: The population frequencies n_{2A} and n_{2B} at the states 2A and 2B respectively as a function of the rate of phenotype switching α, as given in Eq. S3. The frequencies are such that $n_{2A} + n_{2B} = 1$ and the dashed line indicates where $n = 0.5$. The remaining parameters are $\delta = 0.5$ and $d = 0.1$.

VII. DIFFERENT MUTATION PROBABILITIES FOR CELLS IN PHENOTYPE A AND B

So far we have considered all cells in our model to have the same mutation probability μ regardless of which phenotype they are in. However, it has been experimentally observed that cells in secondary phenotypic states often have increased rates of mutation [1, 12]. Figure S7 shows curves of the mean adaptation time $T(\alpha)$ when cells in phenotype B have mutation probabilities (μ_B) greater than that of cells in phenotype A (μ_A). We observe that for low values of α the three curves converge. This is due to α being small enough that successful cells are quicker to evolve by phenotype A than to await the switching events that make phenotype B accessible, irrespective of the value of μ_B. At intermediate α values successful cells travel to the target state primarily by phenotype B, which takes less time the larger μ_B is. Therefore we see lower minimum values of $T(\alpha)$ for larger values of μ_B. Finally, at the largest α values the two phenotype system maps to that of a single phenotype (as discussed later in Section IX C). In this limit, larger mutation probabilities in either phenotype will result in lower adaptation times.

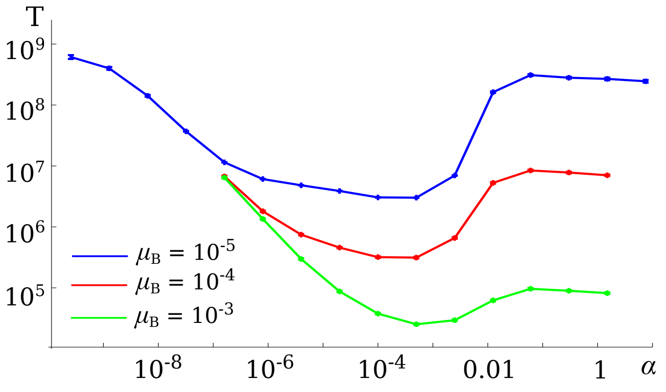

Figure S7: Different mutation probabilities for cells in phenotypes A and B. Shown is a plot of the mean adaptation time T as a function of the rate of phenotypic switching α. The probability of mutation of cells in phenotype A and B are $\mu_A = 10^{-5}$ and μ_B respectively. The remaining parameters are $K = 100$, $\delta = 0.4$ and $d = 0.1$.

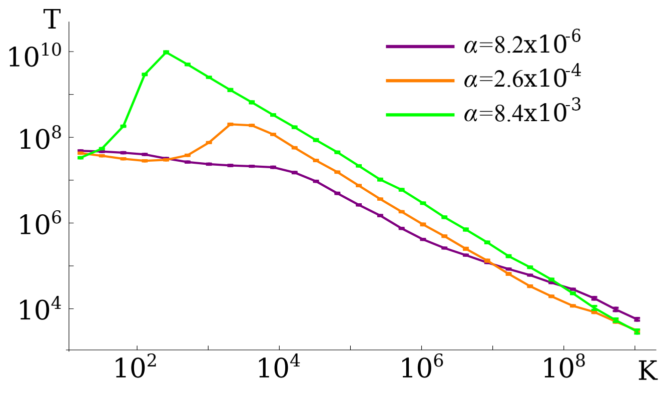

Figure S5: Mean adaptation time as a function of carrying capacity K for different values of α. Remaining parameters are $\mu = 10^{-6}, \delta = 0.4$ and $d = 0.1$

Figure S7 shows that even when $\mu_B \gg \mu_A$ we can still observe a significant valley in the curve of $T(\alpha)$. For example, when $\mu_B = 10\mu_A$ (i.e. the red curve) evolution of cells via phenotype B (i.e. at intermediate α values) remains at least an order of magnitude quicker than in the limits of low and high α. We previously observed in Fig. 2B that the depth of the valley in $T(\alpha)$ increases as the (universal in that case) mutation probability μ decreases. Therefore at the mutation values observed in nature (on average 10^{-10} to 10^{-9} per replication in *E. coli* [2, 7]), we expect the valley in $T(\alpha)$ to be significant enough to withstand cells in phenotype B alone having an increased mutation rate (by orders of magnitude).

VIII. FIXED INCREMENT EVOLUTION OF THE SWITCHING RATE

In the main text, Figure 6 showed simulation results in which newly created cells could mutate their switching rate α by drawing a new value from a discrete set of allowed switching rates. We observed successful cell trajectories to consist largely of those that evolved switching rates to within the optimal range identified in Fig. 2B. Here we consider the same set of allowed α values but, upon mutation of α, the new value is chosen as one of the two closest values to the switching rate of the ancestor cell. This corresponds to α evolving in fixed multiplicative steps, with increase or decrease of α being equally likely. The rest of the algorithm is the same as before.

We again collected trajectories of successful cells in the expanded state space, including states with different α, and calculated transition probabilities between any two states. Fig. S8B shows these probabilities as links of different thickness between the states in state space. We can clearly see some new behaviour here. First, successful trajectories are more likely to feature genotype mutations in phenotype A (more red lines) than in Fig. 6B. Second, the probability of crossing the genotype space at large α (in either phenotype) is much smaller due to the increased number of steps it takes a cell to evolve such large α values. Otherwise, Fig. S8 is similar to Fig. 6. This shows that the details of how mutations of α are implemented do not have a significant effect upon the results of our model.

IX. RANDOM EVOLUTION OF THE SWITCHING RATE

In the main text, Figure 6 showed simulation results when cells could mutate the switching rate α of their offspring by drawing a new value from a discrete set of allowed values. Specifically, Fig. 6C showed a bar chart of the probabilities that genotype space was crossed (combining the mutations from genotype 1 to 2 and those from genotype 2 to 3) for each of the possible switching rates α. In Fig. S9 we consider this situation in more detail, looking at what values of α are most likely to be used by successful cells for the two genetic mutations individually. Figures S9A and S9B show bar charts of the probability that successful cells underwent the genotype mutation 1 to 2 and 2 to 3 respectively for each possible α value. The bars are coloured both red and green to indicate the proportion of the probability that is due to the mutations occurring when the cell is in phenotype A or B respectively. Superimposed on both bar charts is a curve of the mean adaptation time $T(\alpha)$ when alpha is constant, for which the optimal α range can be seen.

We can see in both Figs. S9A and S9B that successful cells are most likely to undergo genetic mutations while in phenotype B (due to the bars in both plots being predominantly green). However, successful cells are more likely to undergo genetic mutations in phenotype A during the mutation from genotype 2 to 3 than from genotype 1 to 2 (due to the larger amount of red seen in the bars in Fig. S9B compared with Fig. S9A). This will be due to the mutation from genotype 2 to 3 being the one that reaches the target genotype (i.e. 3), while the mutation from genotype 1 to 2 results in a cell in phenotype A having to exist at the deleterious valley state.

Figure 6C showed that the most likely α values for successful cells to cross genotype space with were those corresponding to the optimal α range in the curve of the mean adaptation time $T(\alpha)$. In Figs. S9A and S9B we can see that this is also the case when the genetic mutations are considered individually (the peaks of these bar charts correspond with the minimum range in the curve of $T(\alpha)$). This is clearest in Fig. S9B where cells with lower α would take too long to switch back to the correct phenotype and will be outcompeted by those in the optimal α range. It is interesting to note that, as we observed in Fig. 6C, there is almost zero probability that cells with very large α values will be successful in the evolutionary process when α is allowed to evolve. This is due to the weakening of the beneficial effect brought to the process by the existence of the second phenotype, as illustrated mathematically in an earlier section.

X. MATHEMATICAL ANALYSIS

In this section we want to show mathematically the existence of the optimal range for the phenotype switching rate α and find the expression for the mean adaptation time $T(\alpha)$. Due to the complexity of the model, the exact calculation of $T(\alpha)$ is not possible. However, we can find an approximate formula that still agrees very well with numerical simulations while at the same time gives insight into the role of various parameters of the model on the optimal range of α and the dip in $T(\alpha)$.

We first note that, as indicated by computer simulations from Fig. 3B, two trajectories are particularly important in the evolution of a cell of type 3A: that which evolves 3A directly via 1A→2A→3A (represented by the symbol ▁ in Fig. 3B) and that which avoids state 2A entirely (represented by the symbol ⊓). We will refer to these as trajectories A and B respectively (see Fig. S10 for the networks of states these trajectories occur over), with the corresponding mean adaptation times T_A and T_B.

At low values of α, if the valley depth δ is large enough for the optimal α range to exist, successful cells will travel to 3A almost exclusively by trajectories A and B (Fig. 3B). This significantly simplifies the calculation of T: if we treat these two "reaction channels" as occurring independently with rates proportional to $1/T_A$ and $1/T_B$, the mean adaptation time can be approximated by

$$T = \frac{T_A T_B}{T_A + T_B}. \tag{S5}$$

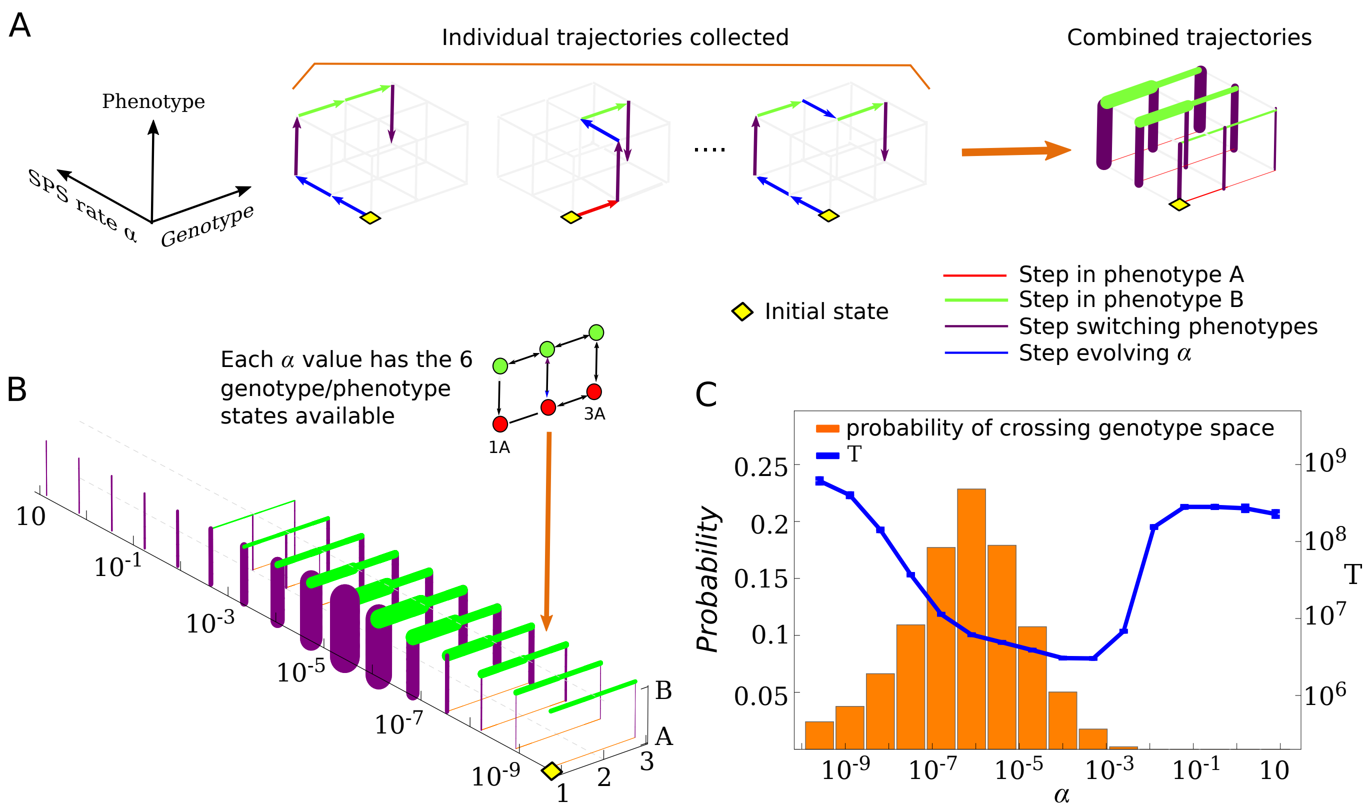

Figure S8: Evolutionary trajectories when α evolves in fixed multiplicative steps. (A) Left: examples of evolutionary trajectories. Upon mutation of α a neighbouring value is selected (with increase or decrease equally likely in the set of allowed values $\alpha = 2.56 \times 10^{-10} \times 5^i$ for $i \in [0, 15]$). (A) Right: trajectories are used to calculate transition probabilities between the states of the system, which are then represented by the thickness of links connecting the states. Red, green and purple links identify steps in phenotype A, phenotype B and between phenotypes respectively. (B) Graph of transition probabilities where line thicknesses are proportional to the probability that a successful trajectory involves that step. The parameters are $K = 100, \mu = 10^{-5}, \delta = 0.4$ and $d = 0.1$, the same that were used in Fig. 2B (blue line). The population begins at the wild-type 1A with $\alpha = 2.56 \times 10^{-10}$ and evolves until a cell in state 3A is produced. (C) The probability that genotype space is crossed at a given α in either phenotype A or B. Superimposed on this plot is the mean adaptation time T as a function of α obtained in a simulation with fixed α (Fig. 2B).

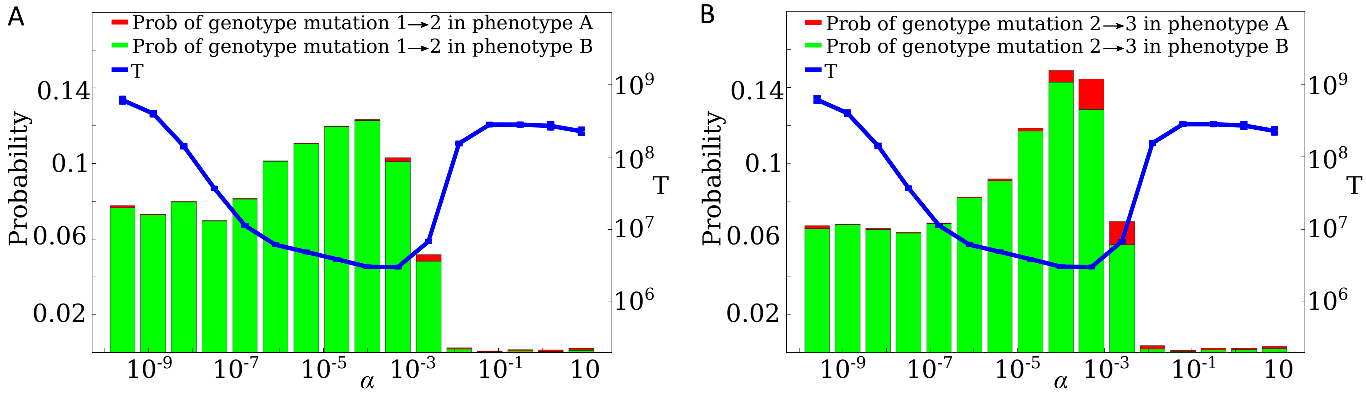

Figure S9: The probability of mutations occurring for different values of α in the trajectories of successful cells, when α can evolve randomly. The population began in state 1A with $\alpha = 2.56 \times 10^{-10}$ and evolved until a cell in state 3A was produced. (A) and (B) show bar charts of the probability that a mutation from genotype 1 to 2 and 2 to 3 respectively occurred for each α value in either phenotype. The bars are in two colours - red and green - to identify what proportion of the mutations took place in phenotype A or B respectively. Superimposed on both bar charts is the same curve of the mean adaptation time $T(\alpha)$ when the model cannot evolve α. Parameters used are $K = 100, \mu = 10^{-5}, \delta = 0.4$ and $d = 0.1$. Results are gathered using a KMC algorithm and averaged over at least 10^4 simulation runs.

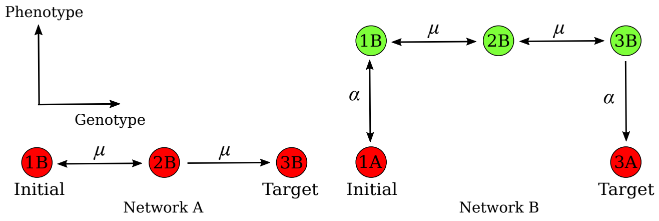

Figure S10: Subnetworks A and B of the full network shown in Fig. 1A. Populations starting at state 1A are constrained to evolve directly to 3A (trajectory A), or by avoiding 2A entirely (trajectory B), with the mean adaptation times T_A and T_B respectively.

Increasing α results in an increase in the variability of trajectories taken by successful cells as indicated by Fig. 3B. However, in the limit of large α we can approximate the dynamics of the system as occurring in a one-dimensional space of states, with A and B phenotypes mixed together as described in Section VI above. This will lead to another formula T_{combined} for the mean adaptation time.

Mathematical descriptions of how asexual populations acquire a sequence of as few as two mutations are already surprisingly complex and exhibit different dynamical regimes [10] depending on parameters such as mutation probability μ and carrying capacity K, as well as the particular fitness landscape being considered. Here we encounter the same difficulty, with the additional complication caused by phenotypic switching. In general, formulas for T_A and T_B will be different for different regimes. Here we shall discuss only those regimes that apply to trajectories from Fig. 3B.

In what follows we shall first show how to calculate T_A, T_B, and T_{combined}. Next, we show how to combine them to obtain a formula for $T(\alpha)$. Finally, we use these formulas to predict the range of optimal α, the minimum T at optimal α, and the phase diagram from Fig. 3B.

A. Low α - stochastic tunnelling via the A trajectory

We assume here that the population size (which is proportional to K) and mutation rate are small enough that the dynamics are stochastic. This is the case for the simulation results presented in Fig. 2B and 3B on which we will focus here. If α is small, the population must evolve via trajectory A. Two regimes are possible in this case: *sequential fixation* [10, 11] and *stochastic tunnelling* [4, 5, 9, 10].

Sequential fixation (SF) occurs when the total rate of mutant production in the population is very small: $\mu dK \ll 1$. If a mutant lineage is to exist long enough to produce the next mutant in the sequence, it is likely that it has become significant in the population (i.e. it has approximately fixed). Otherwise we expect it to be lost due to random drift, particularly if the mutant state is deleterious. In this regime, the mean adaptation time T is dominated by the waiting time for the population to produce mutants that are likely to fix. Therefore we can ignore the intermediate dynamics of the cells, such as drift times (being the time it takes for the mutant population to reach fixation). In this regime, the population remains highly localized and — over large timescales — has the appearance of moving collectively from one state to another upon the event of a mutant fixing.

In the stochastic tunnelling (ST) regime, mutants are produced at greater rates than in the SF regime. Therefore cells are likely to be produced further along a sequence of mutations before cells in the earlier states reach fixation. In general, the mean adaptation time in this regime varies inversely with population size due to the increased rate of mutant production at larger K. The mean adaptation times are smaller than in the SF regime and we find that the drift dynamics of the mutant populations must now be incorporated.

We can show that for the parameters we are interested in here ($K \sim 100, d = 0.1, \delta = 0.4$) the population is unlikely to be in the SF regime. The rate with which mutants are generated at state 2A is approximately $dK(1-d)\mu$ where $K(1-d)$ is the approximate mean number of organisms at state 1A, d is the replication rate (equal to the death rate in the steady state) and μ is the mutation probability. If the probability $P_{2\text{A}}$ of fixation at 2A is sufficiently small, T_A can be approximated by

$$T_A \sim \frac{1}{dK\mu P_{2\text{A}}}. \tag{S6}$$

The probability that a mutant fixes in a state with relative fitness disadvantage δ is [3, 6]

$$P(N,\delta) = \frac{1-e^{\delta}}{1-e^{\delta N}}, \tag{S7}$$

which gives the following formula for $P_{2\text{A}}$:

$$P_{2\text{A}} = \frac{1-e^{\delta}}{1-e^{\delta(1-d)K}}. \tag{S8}$$

Inserting the values $K = 100, d = 0.1, \delta = 0.4, \mu = 10^{-5}$ into Eq. (S6) combined with Eq. (S8) we obtain $T_A \sim 10^{20}$. This is much longer than any T from Fig. 2B, and we shall see below that the alternative regime – stochastic tunnelling – gives a much shorter T_A.

We proceed now with calculating T_A in the stochastic tunnelling (ST) regime. We follow the same approach as in [10] for the general two-step process shown in Fig. S11. The population begins in state $i-1$, at time $t = 0$, and evolves by tunnelling through state i, to produce a cell in the target state $i+1$. We want to calculate the expected time it takes for the first cell to be produced at $i+1$. A mutant at state i that is termed "successful" is one that will go on to produce offspring in state $i+1$. Each mutant at i produces an independent lineage of cells that evolves by a continuous time branching process. All mutants therefore have the same probability of being successful

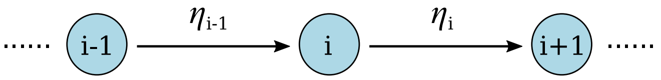

Figure S11: A general two step process to acquire a double mutant. Cells in state $i-1$ can produce mutants in state i, which can produce mutants in state $i+1$. η_j is the rate that a cell in state j produces a mutant in state $j+1$.

(note this would not be the case if states had frequency-dependent fitnesses). We also exclude the possibility of competing mutant lineages at the same state.

For the two-step process in Fig. S11, the expected mean adaptation time T in the stochastic tunnelling regime is

$$T = T_{i-1\to i|\mathrm{suc}} + \tau_i. \tag{S9}$$

Here $T_{i-1\to i|\mathrm{suc}}$ is the expected time it takes the population at $i-1$ to produce the first successful mutant at i, which will dominate T. τ_i is the expected time a successful mutant lineage at state i will drift before producing a cell at state $i+1$. To determine whether a mutant produced at i is successful we need to calculate $P_i(t)$ — the probability that a mutant lineage at i, created at $t=0$, is successful (i.e. produced a cell at $i+1$) by time t. The probability of a mutant at i being at all successful is therefore $P_i = \lim_{t\to\infty} P_i(t)$. Furthermore if we know $P_i(t)$, the drift time τ_i can be calculated by

$$\tau_i = \int_0^\infty \frac{P_i - P_i(t)}{P_i} dt. \tag{S10}$$

Here $P_i - P_i(t)$ is the probability of the mutant not being successful by time t, and the division by P_i ensures that we include only events in which the mutant eventually succeeded. To calculate $P_i(t)$ consider a mutant produced at state i. Let $n(t)$ be the number of mutant cells in this lineage at time t, with initial condition $n(t=0)=1$. The size of the lineage fluctuates in time until it dies out ($n(t)=0$) or produces a cell in the next mutant state $i+1$. In a single realisation of the stochastic process, i.e. one particular trajectory $n(t)$ out of the set of possible trajectories $\{n(t)\}$, the probability that the lineage produces the mutant $i+1$ in time interval $t \to t+dt$ is $\eta_i n(t) dt$, where η_i is the rate with which a cell of type i produces a cell of type $i+1$ (see Fig. S11).

The waiting time for a cell to be created at $i+1$ can be described by an inhomogeneous Poisson process. The probability that a mutant at $i+1$ is created by time t is therefore

$$\begin{aligned} P_i(n(t)) &= 1 - \exp\left(-\int_0^t \eta_i n(t')dt'\right) \\ &= 1 - \exp\left(-\eta_i W(t)\right), \end{aligned} \tag{S11}$$

where $W(t) = \int_0^t n(t')dt'$. If we average (S11) over all possible lineage trajectories $\{n(t)\}$ we get the following expression for $P_i(t)$

$$\begin{aligned} P_i(t) &= \int P_i(n(t)) D[n(t)] \\ &= \int \left[1 - \exp\left(-\eta_i W(t)\right)\right] D[W(t)] \\ &= \int_0^\infty \left(1 - e^{-\eta_i w}\right) P_i(n,w,t) dw dn. \end{aligned} \tag{S12}$$

Here $P_i(n,w,t)$ is the probability density function, such that $P_i(n,w,t)dt$ equals the probability that $W(t)=w$ and $n(t)=n$ during the time interval $(t, t+dt)$.

Let us now introduce $\Phi_i(x,y,t)$ as the Laplace transform of the probability density function $P_i(n,w,t)$:

$$\Phi_i(x,y,t) = \sum_{n=0}^{+\infty} \int_0^\infty P_i(n,w,t) e^{-xn-yw} dw. \tag{S13}$$

If we can determine $\Phi_i(x,y,t)$, then Eq. (S12) allows us to calculate $P_i(t) = 1 - \Phi_i(x=0, y=\eta_i, t)$. The time evolution of $P_i(n,w,t)$ obeys the following equation

$$\begin{aligned} P_i(n,w,t+dt) = {} & \overbrace{(n+1)d_i P_i(n+1, w-ndt, t)dt}^{\text{death}} \\ & + \underbrace{(n-1)b_i P_i(n-1, w-ndt, t)dt}_{\text{birth}} \\ & + \underbrace{\left[1 - n(d_i + b_i)dt\right] P_i(n, w-ndt, t)}_{\text{no change}}. \end{aligned} \tag{S14}$$

Here b_i and d_i are the rates that cells of type i reproduce (i.e. produce offspring of their own type — not mutants) and die respectively. In the limit $dt \to 0$ we obtain the following differential equation for $P_i(n,w,t)$:

$$\begin{aligned} \frac{\partial P_i(n,w,t)}{\partial t} = {} & (n+1)d_i P_i(n+1,w,t) \\ & +(n-1)b_i P_i(n-1,w,t) \\ & -n(d_i+b_i)P_i(n,w,t) - n\frac{\partial P_i(n,w,t)}{\partial w}. \end{aligned} \tag{S15}$$

If we multiply this by $\exp(-xn-yw)$, then integrate over w and sum over n we get

$$\frac{\partial \Phi_i}{\partial t} = \left[-d_i e^x - b_i e^{-x} + (d_i + b_i) + y\right] \frac{\partial \Phi_i}{\partial x}. \tag{S16}$$

This equation can be solved by the method of characteristics to give us

$$\Phi_i(x, y=\eta_i, t) = \frac{\sigma_i^+ (e^{-x} - \sigma_i^-) e^{-b_i(\sigma_i^+ - \sigma_i^-)t} - \sigma_i^- (e^{-x} - \sigma_i^+)}{(e^{-x} - \sigma_i^-) e^{-b_i(\sigma_i^+ - \sigma_i^-)t} - (e^{-x} - \sigma_i^+)}, \tag{S17}$$

where

$$\sigma_i^\pm = \frac{(d_i + \eta_i + b_i) \pm \sqrt{(d_i + \eta_i + b_i)^2 - 4d_i b_i}}{2b_i}. \tag{S18}$$

We can now calculate $P_i(t)$ from (S17):

$$
\begin{aligned}
P_i(t) &= 1 - \Phi_i(x=0, y=\eta_i, t) \\
&= \frac{(1-\sigma_i^+)(1-\sigma_i^-)(e^{-b_i(\sigma_i^+-\sigma_i^-)t}-1)}{\sigma_i^+ - 1 + (1-\sigma_i^-)e^{-b_i(\sigma_i^+-\sigma_i^-)t}}, \qquad \text{(S19)}
\end{aligned}
$$

while the probability of a mutant at i being successful is

$$P_i = \lim_{t\to\infty} P_i(t) = 1 - \sigma_i^-. \qquad \text{(S20)}$$

All terms in Eq. (S9) can now be expressed using Eqs. (S19,S20), giving the mean adaptation time T to be

$$
\begin{aligned}
T &= \overbrace{\frac{1}{\eta_{i-1}N_{i-1}P_i}}^{T_{i-1\to i|\mathrm{suc}}} + \overbrace{\int_0^\infty \left(1 - \frac{P_i(t)}{P_i}\right) dt}^{\tau_i} \\
&= \frac{1}{\eta_{i-1}N_{i-1}(1-\sigma_i^-)} + \frac{1}{b_i(1-\sigma_i^-)} \ln\left[\frac{\sigma_i^+-\sigma_i^-}{\sigma_i^+-1}\right]. \qquad \text{(S21)}
\end{aligned}
$$

Here we have defined the population size at the initial state $i-1$ to be N_{i-1}. All other quantities in (S21), such as $\sigma_i^\pm$ depend only on the replication, death, and mutation rates, and not the population size.

We will now apply this method to calculate the mean adaptation time T_A for trajectory A in Fig. S10. The mean adaptation time T_A is given by Eq. (S21), in which $i-1$ corresponds to state 1A and i to state 2A. Therefore we have that

$$T_A = T_{1\mathrm{A}\to 2\mathrm{A}|\mathrm{suc}} + \tau_{2\mathrm{A}}, \qquad \text{(S22)}$$

in which (identifying the values of the variables in our model to use in Eq. (S21)):

$$
\begin{aligned}
N_{i-1} &= N_{1A} \approx (1-d)\,K, && \text{(S23)}\\
\eta_{i-1} &= \eta_{1A} = \left(1 - \frac{N_T}{K}\right)\mu r_{1A} \approx \mu d, && \text{(S24)}\\
b_i = b_{2A} &= \left(1 - \frac{N_T}{K}\right)(1-2\mu) r_{2A} \\
&\approx (1-\delta)\, d(1-2\mu), && \text{(S25)}\\
d_i &= d, && \text{(S26)}
\end{aligned}
$$

where we have used that $r_{1\mathrm{A}} = 1, r_{2\mathrm{A}} = (1-\delta)$ and that most of the population is expected to remain in state 1A in this regime, so $N_T \approx N_{1A}$ which is approximately given by Eq. (S23). This approximation means that the logistic factor controlling growth is $(1 - N_T/K) \approx d/r_{1\mathrm{A}} = d$. Substituting these values into Eq. (S21) gives us

$$
\begin{aligned}
T_{1\mathrm{A}\to 2\mathrm{A}|\mathrm{suc}} &= \frac{1}{\left(1 - \frac{d}{r_{1\mathrm{A}}}\right) K d\mu (1-\sigma_{2\mathrm{A}}^-)}, && \text{(S27)}\\
\tau_{2\mathrm{A}} &= \frac{r_{1\mathrm{A}} \ln\left[\frac{\sigma_{2\mathrm{A}}^+-\sigma_{2\mathrm{A}}^-}{\sigma_{2\mathrm{A}}^+-1}\right]}{r_{2\mathrm{A}} d(1-2\mu)(1-\sigma_{2\mathrm{A}}^-)}, && \text{(S28)}
\end{aligned}
$$

and from (S18) we know that

$$\sigma_{2\mathrm{A}}^\pm = \frac{r_{1\mathrm{A}} + r_{2\mathrm{A}}(1-\mu) \pm \sqrt{(r_{1\mathrm{A}} + r_{2\mathrm{A}}(1-\mu))^2 - 4 r_{1\mathrm{A}} r_{2\mathrm{A}}(1-2\mu)}}{2 r_{2\mathrm{A}}(1-2\mu)}. \qquad \text{(S29)}$$

Since in the limit we are interested here ($K\mu \ll 1$), $T_{1\mathrm{A}\to 2\mathrm{A}|\mathrm{suc}}$ is much larger than $\tau_{2\mathrm{A}}$ we can neglect the last term in Eq. (S22) and write the expresion for T_A in the stochastic tunnelling regime:

$$T_{\mathrm{A,ST}} \approx \frac{1}{(1-d)\,K d\mu^2 (1/\delta - 1)}, \qquad \text{(S30)}$$

where we have replaced $\sigma_{2\mathrm{A}}^-$ by $1-\mu+\mu/\delta$ which is valid in the limit of small μ considered here. As expected, T_A from Eq. (S30) is inversely proportional to μ^2 since it requires two mutation steps to reach 3A from 1A. Inserting $K = 100, d = 0.1, \delta = 0.4, \mu = 10^{-5}$ into Eq. (S30) we obtain $T_A \approx 7.4 \times 10^8$, which is orders of magnitude smaller than the value predicted via the sequential fixation formula (S6).

B. Intermediate α - sequential fixation via the B trajectory

As we increase α, the probability of switching to phenotype B increases and the dynamics of the system is well described by neutral evolution along trajectory B. As before, we consider the small $K\mu$ and $K\alpha$ limit. This corresponds to the sequential fixation regime mentioned earlier and enables us to approximate the dynamics by the whole population jumping between the sites of trajectory B.

In what follows we neglect population extinction which is very unlikely for $K \gg 10$ considered here. The population performs a continuous time, 1D asymmetric random walk, with site-dependent transition rates. The mean time it takes a walker to go from site 0 to M (in which 0 is a reflecting boundary) is [8]

$$t_{(0,M)} = \sum_{k=0}^{M-1} \frac{1}{p_k} + \sum_{k=0}^{M-2} \frac{1}{p_k} \sum_{i=k+1}^{M-1} \prod_{j=k+1}^{i} \frac{q_j}{p_j}. \qquad \text{(S31)}$$

Here p_i and q_i are the rates that in each timestep the walker moves from site i to $i+1$ and $i-1$ respectively.

We can use Eq. (S31) to calculate T_B by identifying states $i = 0,1,2,3,4$ ($M = 4$) with our states 1A, 1B, 2B, 3B, and 3A in Fig. S10. We therefore have that

$$
\begin{aligned}
T_B &= \frac{\frac{q(3)q(2)}{p(2)p(3)} + \frac{q(2)}{p(2)}}{p(1)} + \frac{\frac{q(2)q(1)}{p(1)p(2)} + \frac{q(2)q(3)q(1)}{p(1)p(2)p(3)} + \frac{q(1)}{p(1)}}{p(0)} \\
&\quad + \frac{q(3)}{p(2)p(3)} + \frac{1}{p(0)} + \frac{1}{p(1)} + \frac{1}{p(2)} + \frac{1}{p(3)}, \qquad \text{(S32)}
\end{aligned}
$$

where the rate $p(0)$ corresponds to the transition from 1A to 1B, rate $p(1)$ to the transition 1B→2B, etc., while the rate $q(1)$ corresponds to the transition 1B→1A, rate $q(2)$ to 2B→1B, and so on. These rates are given by the rate with which a mutant/opposite phenotype is generated

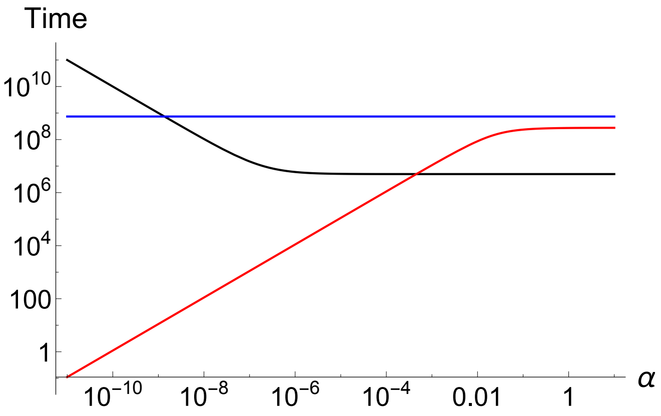

Figure S12: Mean adaptation time as predicted in different regions of the switching rate α: Eq. (S30) (blue line), Eq. (S36) (black line), and Eq. (S40) (red line). Parameters are $K = 100, \mu = 10^{-5}, d = 0.1, \delta = 0.4$. The point of intersection between the blue and black lines corresponds to α^*.

(equal to $\mu K(1-d)$ and $\alpha K(1-d)$, respectively), times the fixation probability in the new state $(1/(K(1-d))$ since evolution is neutral and all states have the same growth rate). The rates are

$$p(0) = q(1) = \alpha, \tag{S33}$$
$$p(1) = q(2) = p(2) = q(3) = d\mu, \tag{S34}$$
$$p(3) = \alpha K(1-d). \tag{S35}$$

Inserting the above rates into Eq. (S32) we obtain a simple expression for T_B in the sequential fixation regime,

$$T_{\mathrm{B,SF}} = \frac{1}{\alpha} + \frac{5}{d\mu} + \frac{4}{\alpha K(1-d)} \approx \frac{1}{\alpha} + \frac{5}{d\mu}, \tag{S36}$$

where we have neglected the last term as being much smaller than the two other terms.

Figure S12 compares T_B from Eq. (S36) and T_A from Eq. (S30). While it takes much longer for the population to travel along trajectory B than A for very small α, the mean time to adaptation T_B decreases inversely proportional to α and becomes smaller than T_A at some α_*. To find α^*, we equate T_A and T_B from Eqs. (S30,S36):

$$\frac{1}{(1-d)\,Kd\mu^2(1/\delta - 1)} = \frac{1}{\alpha^*} + \frac{5}{d\mu}, \tag{S37}$$

from which we obtain that

$$\alpha^* \cong Kd\mu^2. \tag{S38}$$

C. Large α - the combined trajectory

In the limit of very fast phenotypic switching (large α) we have the situation described in Section VI: the 6-state system reduces to 3 states which now combine phenotypes A and B. Two of the states (1 and 3) have the same growth rates, $r_{1,\mathrm{comb}} = r_{3,\mathrm{comb}} = 1$ (as we are only interested only in the creation of the first cell at state 3A in the 6 state system). The growth rate of the middle state is

$$r_{2,\mathrm{comb}} = n_{2\mathrm{A}} r_{2\mathrm{A}} + n_{2\mathrm{B}} r_{2\mathrm{B}} = \frac{(d+\alpha)(2-\delta) + \sqrt{\delta^2(\alpha+d)^2 - 4\alpha^2(\delta-1)}}{2(2\alpha+d)}, \tag{S39}$$

where we have used Eq. (S3) for $n_{2\mathrm{B}}$. Since the combined system has the same structure as trajectory A from Section X A, the system is again in the stochastic tunnelling regime and we can use Eq. (S30) to calculate the mean adaptation time. We only need to replace the depth δ of the valley by the new effective depth $1 - r_{2,\mathrm{comb}}$. This gives

$$T_{\mathrm{comb,ST}} \approx \frac{1}{(1-d)\,Kd\mu^2(1/(1-r_{2,\mathrm{comb}}) - 1)}. \tag{S40}$$

Equation (S40) is valid only if the fitness valley is sufficiently deep. For shallow valleys ($1 - r_{2,\mathrm{comb}} \lesssim 1/K$) the dynamics becomes neutral and is better approximated by the sequential fixation formula. The characteristic α_+ when this happens can be determined from the equation $1 - r_{2,\mathrm{comb}}(\alpha_+) = 1/K$ which gives

$$\alpha_+ \approx d/K. \tag{S41}$$

D. Comparison to simulations

Figure 3C shows the predicted mean time to adaptation as a function of α. The small and intermediate α formulas have been combined together by means of Eq. (S5) whereas the large α solution (S40) has been plotted separately. The agreement between the analytics and computer simulations is very good, especially for low α values. The position of the optimal α region is also predicted with good accuracy despite the many approximations that we have made.

E. The optimal range of α, the minimum $T(\alpha)$, and the phase diagram

To find the small-α boundary of the optimal α regime, we notice in Eq. (S36) that T_B reaches a plateau when $\alpha \gg \alpha_-$, for which

$$\alpha_- = d\mu. \tag{S42}$$

Therefore α_- is the lower boundary of the dip in $T(\alpha)$. The upper boundary α_+ is defined by Eq. (S41). The

minimum of the mean adaptation time is given by the minimum of $T_{\mathrm{B,ST}}$ from Eq. (S36):

$$T_{\min} \approx 5/(d\mu). \tag{S43}$$

These mathematical results can be used to understand the three regions seen in Fig. 3B from the main text:

- For $\alpha \ll \alpha^* = Kd\mu^2$, the population evolves along trajectory A and does not switch phenotypes.
- For $Kd\mu^2 \ll \alpha \ll \alpha_- = d\mu$, switching becomes more important and trajectory B dominates.
- For $d\mu \ll \alpha \ll \alpha_+ = d/K$, the system is in the optimal switching regime. The population evolves almost exclusively along trajectory B, avoiding fitness valley at state 2A.
- For $\alpha \gg d/K$ phenotypic switching mixes phenotypes A and B. The population evolves along a complicated trajectory that includes both phenotypes.

The boundaries of different regions predicted by the above analytic calculation are in good numerical agreement with the diagram in Fig. 3B for $K = 100, \delta = 0.4, d = 0.1$. In particular, the optimal switching region vanishes for $\mu \gg 0.01$ as predicted by the equality $\alpha_* = \alpha_+$ which predicts that $\mu_{\mathrm{critical}} \propto 1/K$.

We note that all calculations presented in this section are not valid when the population size K or the mutation probability μ are very large. In this case the population may be in the deterministic or semi-deterministic regime, and different formulas will apply.

[1] Bos, J., Q. Zhang, S. Vyawahare, E. Rogers, S. M. Rosenberg, and R. H. Austin. "Emergence of antibiotic resistance from multinucleated bacterial filaments." *Proceedings of the National Academy of Sciences of the United States of America* 112, 1: (2015) 178–83.

[2] Drake, J. W. "Rates of spontaneous mutation among RNA viruses." *Proceedings of the National Academy of Sciences of the United States of America* 90, 9: (1993) 4171–4175.

[3] Ewens, W. J. *Mathematical population genetics.* Springer, 2004, second edition.

[4] Gillespie, J. "Molecular evolution over the mutational landscape." *Evolution* 38, 5: (1984) 1116–1129.

[5] Iwasa, Y., F. Michor, and M. a. Nowak. "Stochastic tunnels in evolutionary dynamics." *Genetics* 166, 3: (2004) 1571–9.

[6] Kimura, M. "On the probability of fixation of mutant genes in a population." *Genetics* 47, 391: (1962) 713–719.

[7] Lee, H., E. Popodi, H. Tang, and P. L. Foster. "Rate and molecular spectrum of spontaneous mutations in the bacterium Escherichia coli as determined by whole-genome sequencing." *Proceedings of the National Academy of Sciences* 109, 41: (2012) E2774–E2783.

[8] Murthy, K., and K. Kehr. "Mean first-passage time of random walks on a random lattice." *Physical Review A* 40, 4: (1989) 2082–2087.

[9] Weinreich, D. M., and L. Chao. "Rapid evolutionary escape by large populations from local fitness peaks is likely in nature." *Evolution; international journal of organic evolution* 59, 6: (2005) 1175–82.

[10] Weissman, D. B., M. M. Desai, D. S. Fisher, and M. W. Feldman. "The rate at which asexual populations cross fitness valleys." *Theoretical population biology* 75, 4: (2009) 286–300.

[11] Wright, S. "Evolution in Mendelian populations." *Genetics* 16: (1931) 97–159.

[12] Zhang, Q., G. Lambert, D. Liao, H. Kim, K. Robin, C.-k. Tung, N. Pourmand, and R. H. Austin. "Acceleration of Emergence of Bacterial Antibiotic Resistance in Connected Microenvironments." *Science* 333, 2011: (2011) 1764–1767.
